# Combined Anterior Cruciate Ligament Reconstruction (ACLR) and Lateral Extra-articular Tenodesis through the Modified Lemaire Technique versus Isolated ACLR: A Meta-analysis of Clinical Outcomes

**DOI:** 10.1055/s-0044-1785492

**Published:** 2024-04-10

**Authors:** Essy Dwi Damayanthi, Erica Kholinne, Leonard Christianto Singjie, Muhammad Sakti, Ira Juliet Anesstesia

**Affiliations:** 1 Departamento de Ortopedia e Traumatologia, Ulin General Hospital, Faculty of Medicine, Lambung Mangkurat University, Banjarmasin, Indonésia; 2 Departamento de Cirurgia Ortopédica, St. Carolus Hospital, Faculty of Medicine, Universitas Trisakti, Jacarta, Indonésia; 3 Departamento de Cirurgia Ortopédica, Faculty of Medicine, Hasanuddin University, Macáçar, Indonésia

**Keywords:** anterior cruciate ligament reconstruction, joint instability, knee joint, tenodesis, treatment outcome

## Abstract

**Objective** Lateral extra-articular tenodesis (LET) has been proposed to resolve rotatory instability following anterior cruciate ligament reconstruction (ACLR). The present meta-analysis aimed to compare the clinical outcomes of ACLR and ACLR with LET using the modified Lemaire technique.

**Materials and Methods** We performed a meta-analysis following the Preferred Reporting Items for Systematic Reviews and Meta-Analysis (PRISMA) staement. The literature search was performed on the PubMed, EBSCOHost, Scopus, ScienceDirect, and WileyOnline databases. The data extracted from the studies included were the study characteristics, the failure rate (graft or clinical failure) as the primary outcome, and the functional score as the secondary outcome. Comparisons were made between the patients who underwent isolated ACLR (ACLR group) and those submitted to ACLR and LET through the modified Lemaire technique (ACLR + LET group).

**Results** A total of 5 studies including 797 patients were evaluated. The ACLR + LET group presented a lower risk of failure and lower rate of rerupture than the ACLR group (risk ratio [RR] = 0.44; 95% confidence interval [95%CI]: 0.26 to 0.75; I ^2^ = 9%; *p* = 0.003). The ACLR + LET group presented higher scores on the Knee Injury and Osteoarthritis Outcome Score (KOOS) regarding the following outcomes: pain, activities of daily living (ADL), sports, and quality of life (QOL), with mean differences of 0.20 (95%CI: 0.10 to 0.30; I ^2^ = 0%; *p* < 0.0001), -0.20 (95%CI: -0.26 to -0.13; I ^2^ = 0%; *p* < 0.00001), 0.20 (95%CI: 0.02 to 0.38; I ^2^ = 0%; *p* = 0.03), and 0.50 (95%CI: 0.29 to 0.71; I ^2^ = 0%; *p* < 0.00001) respectively when compared with the ACLR group.

**Conclusion** Adding LET through the modified Lemaire technique to ACLR may improve knee stability because of the lower rate of graft rerupture and the superiority in terms of clinical outcomes.

**Level of Evidence** I.

## Introduction

Anterior cruciate ligament (ACL) ruptures are among the most commonly studied injuries in orthopedic research, and their incidence is estimated to range from 30 to 78 cases per 100 thousand people a year. 1 After ACL reconstruction (ACLR), 61% to 89% of athletes successfully return to sports, typically between 8 and 18 months after the reconstruction, depending on the level of play. 1 Under certain conditions, a rerupture can occur, which may be devastating. The reported rate of ACL rerupture ranges from 1% to 11%, and they may be caused by traumatic reinjuries, biological graft failure, or technical surgical errors. 1 2

The management of ACL injury in patients at a higher risk of rerupture remains controversial. It has been shown that the risk factors for graft rupture include younger patients (< 20 years of age), those with generalized hypermobility and physiologic knee hyperextension, and those returning to high-risk (pivoting) sports. 3 Further, Saita et al. 4 showed that knee hyperextension and a small lateral condyle are associated with greater anterolateral rotatory instability, which is difficult to manage in patients who continue to show a positive pivot shift after isolated ACLR. In the literature, 3 5 6 7 the MacIntosh, Lemaire, and anterolateral ligament (ALL) reconstruction techniques have been shown to resolve anterolateral rotatory instability. Reconstruction of the ALL was found to reduce the graft failure rate in large series of patients at 2 years of follow-up. 8 The modified Lemaire technique has been shown to present a low complication rate and to cause a reduction in pivot-shift instability. 6

One of the reasons to favor lateral extra-articular tenodesis (LET) rather than ALL reconstruction is because of the evidence indicating that ALL reconstruction could overconstrain the lateral joint while not being as mechanically advantageous in resisting rotation. 9 10 The aim of LET is to decrease the rerupture rate by providing more stability to the knee joint. A cohort study by Cavaignac et al. 11 reported that ACLR with LET showed better graft maturity on magnetic resianace imaging (MRI) scans after one year of the procedures. Mayr et al. 12 focused on the modified Lemaire technique, which has recently been used to perform LET, and they showed that it may decrease the strain on the graft as well as residual rotational laxity, thus improving the clinical outcomes. Therefore, we conducted a meta-analysis to determine the impact of ACLR and LET through the modified Lemaire technique compared with ACLR on patients with ACL rupture in terms of the rerupture rate and clinical outcome. The objective of the present study was to determine the surgical outcome of ACLR with modified Lemaire LET for ACL rupture, which is be represented by the rerupture rate and clinical outcomes.

## Materials and Methods

### Search Strategy

We conducted a systematic review and meta-analysis based on the Preferred Reporting Items for Systematic Reviews and Meta-Analysis (PRISMA) statement. 13 The study protocol was registered in the Open Science Framework. The literature search was conducted in June 2022 on several databases, including PubMed, EBSCOHost, Scopus, ScienceDirect, and WileyOnline, focusing on the Population, Intervention, Control, and Outcome (PICO) strategy. The population consisted of patients with ACL tears, the intervention was ACLR and LET through the modified Lemaire technique, and isolated ACLR was the comparator. The outcomes assessed were the rerupture rate as the primary outcome, and the patient-reported outcome measures (PROMs) and functional scores as secondary outcomes.

### Study Selection

The exclusion criteria were animal studies, revision cases of ACLR, concomitant posterior cruciate ligament (PCL) or meniscus reconstruction, underlying congenital condition or neoplasm, ACLR with ALL reconstruction, patients treated with pharmacologic treatment, nutrition treatment, physical therapy or isolated rehabilitation, and ACLR with LET not through the modified Lemaire technique. Only studies published in English within the last twenty years were included.

### Quality Appraisal and Risk of Bias Assessment

Two authors (ED and LC) performed the identification and selectionof studies, as well as data extraction. The quality assessment was performed by two other authors (MS, IJA). Differences in opinion between the two reviewers were resolved by reassessment and discussion with another author (EK). The selected studies were assessed using the Joanna Briggs Institute's tools for critical appraisal. 14

### Data Extraction and Analysis

The data extracted from the included studies were characteristics such as author and year of publication, location, design, sample characteristics (age, gender, injury type), failure (graft or clinical failure), and outcome (Knee Injury and Osteoarthritis Outcome Score [KOOS], functional outcome, and clinical outcome). The studies were assessed qualitatively and quantitatively using the Review Manager (RevMan, The Cochrane Collaboration, London, United Kingdom) software, version 5.4. The random-effects model was used to calculate pooled ratio from each study based on the heterogeneity. The Cochrane I-squared (I ^2^ ) test was conducted to determine the heterogeneity. The results of the studies are presented in a forest plot with the pooled risk ratio (RR).

## Results

**Fig. 1:**
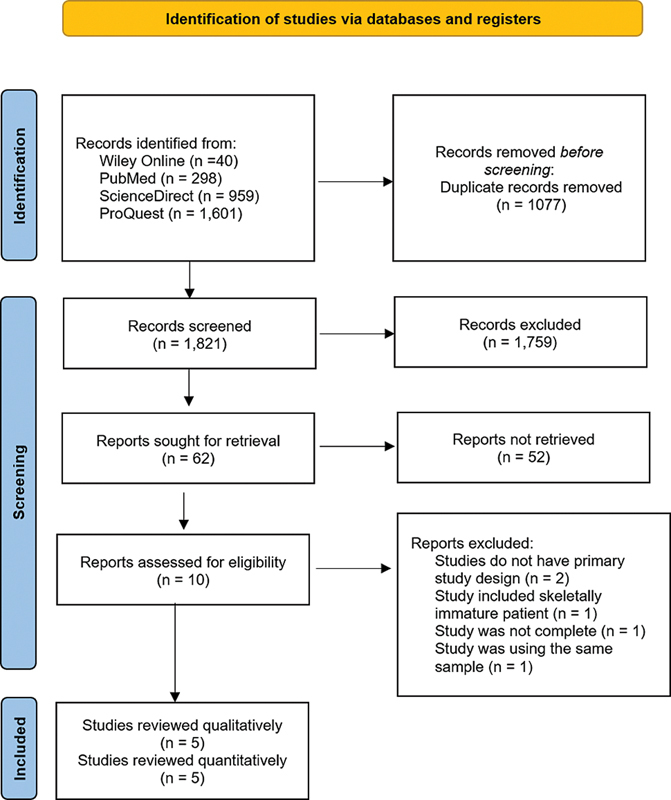
PRISMA flowchart.

In the initial screening, 163 studies were retrieved ( Fig. 1 ). Among the ten remaining studies, two did not have a primary outcome (success rate), 12 15 one included skeletally-immature patients, 16 and one did not have adequate control. 17 In the end, we found five studies 18 19 20 21 22 eligible for qualitative and quantitative analysis after the searching strategies were applied. Two studies were randomized controlled trials (RCTs) 18 19 and three were cohort studies. 20 21 22

The appraisal of the studies using the Joanna Briggs Institute's critical appraisal tools showed that all of them were considered good in terms of methodological quality and lack of the possibility of bias in their design, conduct and analysis ( Table 1 ). Table 2 shows the characteristics of the studies. including the intraoperative details. Table 3 shows the outcome parameters measured for each study.

**Table 1:** Critical appraisal the results of the selected studies

Study	Items on the Joanna -Briggs Institute's tools for critical appraisal												
	1	2	3	4	5	6	7	8	9	10	11	12	13
Castoldi et al. 18 (2020)	Y	Y	Y	NA	NA	Y	Y	Y	Y	Y	Y	Y	Y
Getgood et al. 19 (2020)	Y	Y	Y	NA	NA	Y	Y	Y	Y	Y	Y	Y	Y
Eggeling et al. 21 (2022)	Y	Y	Y	NA	NA	Y	Y	Y	Y	Y	Y	Y	Y
Rowan et al. 22 (2019)	Y	Y	Y	Y	Y	Y	Y	Y	Y	Y	Y		
Dejour et al. 20 (2013)	Y	Y	Y	NA	NA	Y	Y	Y	Y	Y	Y		

Abbreviations: NA, not available; Y, yes.

**Table 2:** Characteristics of the included studies

Study	Type	Level of evidence	No. of patients						Mean Age (years)		Mean/ minimum follow-up (years)	Mean Time from Injury to Surgery (months)		Graft		Tensioning method	LET fixation	ACL fixation
			LET			Non-LET			LET	Non-LET		LET	Non-LET	ACL	LET			
			M	F	T	M	F	T										
Castoldi et al. 18 (2020)	RCT	2	47	13	60	43	18	61	26.2	25.9	19.4	NR	NR	BPTB	G	30 degrees of knee flexion	Suture fixation	Femoral/tibial side: absorbable interference screw
Getgood et al. 19 (2020)	RCT	2	151	155	306	151	161	312	19.1	18.8	2	9.3	8.1	H	ITB	60–70 degrees of knee flexion	Barbed Richards fixation staple	Femoral side: femoral cortical suspensory fixation; tibial side: tibial interference screw
Eggeling et al. 21 (2022)	C	3	13	10	23	35	20	55	33.3	31.9	2.4	NR	NR	BPTB, H, Q	ITB	45 degrees of knee flexion	Suture fixation	Femoral/tibial sides: interference screw
Rowan et al. 22 (2019)	C	3	34	21	55	120	98	218	26	33	2	NR	NR	ST + G	ITB	30 degrees of knee flexion	Fixation staple with spike	Femoral/tibial sides: absorbable interference screw
Dejour et al. 20 (2012)	C	3	20	5	25	17	8	25	21.4	27.5	2	10.78	12.96	BPTB	G	Full extension	Suture fixation	Femoral/tibial sides: interference screw

**Abbreviations:** ACL, anterior cruciate ligament; BPTB, bone-patellar tendon-bone; C, cohort; F, female; G, gracilis; H, hamstring; ITB, iliotibial band; LET, lateral extra-articular tenodesis; M, male; NR, not reported; Q, quadriceps; RCT, randomized controlled trial; ST, semitendinosus; T, total.

**Table 3:** Outcomes of the included atudies

Study	Failure (%LET vs. %control)	Outcome								
		KOOS pain score	KOOS symptom score	KOOS ADL score	KOOS sports score	KOOS QoL score	Tegner score	International Knee Documentation Committee score	Lysholm score	Marx score
Castoldi et al. 18 (2020)	Graft failure: 5/38 (13%) vs. 12/42 (29%)	−	−	−	−	−	−	82.4 ± 11.2 vs. 81.1 ± 14.38	−	−
Getgood et al. 19 (2020)	Clinical failure: 72/291 (25%) vs. 120/298 (40%) (RR: 0.38; 95%CI: 0.21–0.52; *p* = 0.0001)	92.1 ± 0.6 vs. 91.9 ± 0.6	84.7 ± 0.8 vs. 84.6 ± 0.8	97 ± 0.4 vs. 97.2 ± 0.4	85.3 ± 1.1 vs. 85.1 ± 1.1	63.8 ± 18.9 vs. 58.4 ± 19.7	−	87.3 ± 0.8 vs. 86.8 ± 0.8	−	12.1 ± 5.5 vs. 12.7 ± 4.7
	Graft ailure: 11/291 (4%) vs. 34/298 (11%) (RR: 0.67; 95%CI, 0.36–0.83; *p* = 0.001)						−	−	−	−
Eggeling et al. 21 (2022)	Clinical failure: 3/23 (13%) vs. 6/55 (10.9%)	87.9 ± 14.6 vs. 87.9 ± 14.1	87.6 ± 15.4 vs. 87.3 ± 14.8	95.2 ± 8.2 vs. 93 ± 10	72.6 ± 25.9 vs. 76 ± 22.7	75.4 ± 1.3 vs. 74.9 ± 1.3	5.7 ± 1.3 vs. 5.9 ± 1.5	80.1 ± 14.9 vs. 77.5 ± 16.2	81.9 ± 14.2 vs. 83.8 ± 14.5	−
Rowan et al. 22 (2019)	Clinical failure: 0/46 (0%) vs. 13/125 (10.4%)	−	−	−	−	−	8.04 ± 1.35 vs. 75.4 ± 1.35	−	98 ± 11.2 vs. 90 ± 16.2	−
Dejour et al. 20 (2012)	Clinical failure: 0/25 (0%) vs. 2/25 (8%)	−	−	−	−	−	−	−	−	−

**Abbreviations:** 95%CI, 95% confidence interval; ADL, activities of daily living; KOOS, Knee Injury and Osteoarthritis Outcome Score; LET, lateral extra-articular tenodesis; QoL, quality of life; RR, risk ratio; vs., versus.

**Fig. 2:**
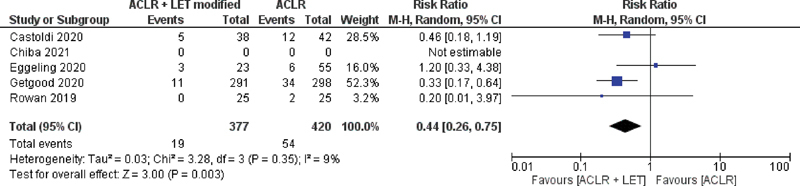
Risk ratio for failure in the group of ACLR + LET through the modified Lemaire technique and the ACLR group.

In the present study, we found that the RR for failure was lower in the ACLR + LET group with the modified Lemaire rechnique than in the ACLR group, with low heterogeneity among the studies (RR = 0.44; 95% confidence interval [95%CI]: 0.26 to 0.75; I ^2^ = 9%; *p* = 0.003) ( Fig. 2 ).

**Fig. 3:**
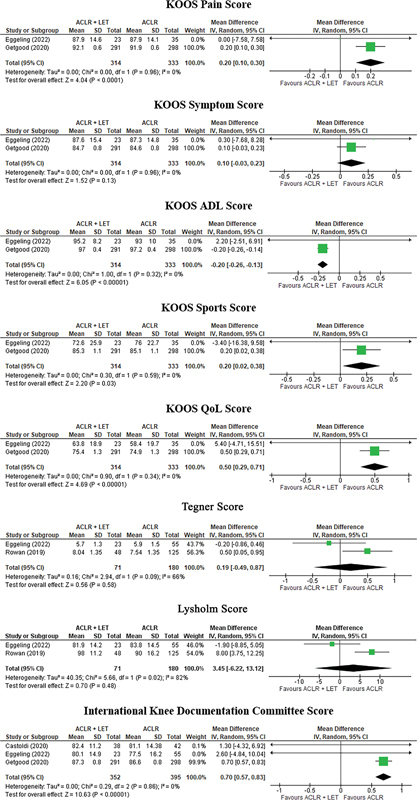
Forest Plot of the secondary outcome of the included studies.

The meta-analysis showed a superiority of the ACLR + LET group with the modified Lemaire Technique regarding of the following outcomes on the KOOS: pain, activities of daily living (ADL), sports, and quality of life (QoL), with mean differences of 0.20 (95%CI: 0.10 to 0.30; *p* < 0.0001), -0.20 (95%CI: -0.26 to -0.13; *p* < 0.00001), 0.20 (95%CI: 0.02 to 0.38; *p* = 0.03) and 0.50 (95%CI: 0.29 to 0.71; *p* < 0.00001) respectively. However, there was no significant difference between the groups in the symptom scores on the KOOS, with a mean difference of 0.10 (95%CI: -0.03 to 0.2; *p* = 0.13). Neither were there were differences between the groups regarding the scores on the Tegner Activity Scale (TAS) and Lysholm Knee Scoring Scale (LKSS), with mean differences of 0.19 (95%CI: -0.49 to 0.87; *p* = 0.58) and 3.45 (95%CI: -6.22 to 13.22; *p* = 0.48) respectively. However, there was a significant difference regarding the scores on the International Knee Documentation Committee (IKDC) Subjective Knee Form, with a mean difference of 0.70 (95%CI: 0.57 to 0.83; *p* < 0.00001). Low heterogeneity was found in the scores on the KOOS and IKDC Subjective Knee Form , but high heterogeneity was found in TAS and LKSS scores. ( Fig. 3 ).

## Discussion

The most important findings of the current research were that, when compared with the ACLR group, the ACLR + LET with modified Lemaire presented a lower failure rate and significant superiority regarding the functional outcome based on the mean differences in pain, ADL, sports, and QoL domains.

When compared with the ACLR group, the ACLR + LET with modified Lemaire group was found to present a lower failure rate (RR = 0.44; I ^2^ = 9%; *p* = 0.003). The ACLR + LET with modified Lemaire group showed a significant superiority regarding the functional outcome based on the mean differences in the scores on the KOOS domains of pain, ADL, sports, and QoL ( *p* < 0.00001; *p* < 0.03; *p* < 0.00001; and *p* < 0.00001 respectively) and the scores on the IKDC Subjective Knee Form ( *p* < 00001).

Rotational stability was not recovered with isolated ACLR in a certain population. 23 Therefore, both intra- and extra-articular procedures were necessary to improve ACL stability, thus improving the ability to perform sports in this population. It is known that LET is one of the extra-articular procedures that preserves knee stability. Na et al. 23 compared isolated ACLR to ACLR combined with anterolateral extra-articular procedures, and they noticed that both techniques improved pivot-shift grades and graft failure rates. However, in the ACLR + LET group, there was an increased risk of knee stiffness and adverse events. 23 These findings explain the significantly better KOOS and IKDC scores in the group submitted to ACLR + LET with the modified Lemaire technique.

Various LET procedures, namely Lemaire, MacIntosh, and ALL reconstruction, are the choices to manage rotatory instability. However, a in a kinematic study published by Inderhaug et al. 10 in 2017, the authors found that ALL reconstruction is underconstrained procedure. Compared with ALL reconstruction, the modified Lemaire technique has been shown to present a low complication rate and to cause a reduction in pivot-shift instability. The modified Lemaire technique also showed good graft survival and PROMs in a high-risk population. ^1^ This may suggest that LET is an effective technique to restore joint stability to a knee with additional features of laxity. 2 10

In a meta-analysis, Onggo et al. 24 compared ACLR and ACLR + LET through any method, and the inclusion of studies with a minimum of two years of follow-up. They found improved stability (RR = 0.59; 95%CI: 0.39 to 0.88) and improved clinical outcomes in the ACLR + LET group, shown by mean differences in the IKDC and Lysholm scores of 2.31 (95%CI: 0.54 to 4.09) and 2.71 (95%CI 0.68 to 4.75) respectively. In addition, there was less likelihood of graft rerupture in the ACLR + LET group, with an RR of 0.31 (95%CI: 0.17 to 0.58). 24 In a single-armed systematic review involving 851 patients who underwent ACLR + LET, Grassi et al. 25 showed favorable results in terms of KOOS scores, with 74% of the patients returning to their previous sports activities, as well as complication and failure rates of 8.0% and 3.6% respectively.

The combination of ACLR and LET has also been considered safe for the patients. Feller et al. 26 reported that, at the 12-month follow-up, a contact-related graft rupture occurred in one patient, accounting for 4% of the total. Two additional ACL injuries in the opposite knee were observed, making up 9% of the cases, with 1 of them being an ACL graft rupture at 11 months postoperatively and another occurring at 22 months. Furthermore, a separate incident of contralateral ACL graft rupture took place at the 26-month follow-up. 26 Concerns were raised about the potential for excessive restriction of the lateral compartment of the knee and the subsequent development of lateral compartment osteoarthritis in relation to LET. However, a meta-analysis by Devitt et al. 27 provided strong evidence that the addition of LET reduces the movement of the lateral compartment. Biomechanical studies support these clinical findings, showing that both anatomic ALL reconstruction and LET procedures can overly restrict the lateral compartment. On the contrary, a recent systematic review indicated that adding LET to ACLR does not increase long-term osteoarthritis rates. While there is insufficient evidence to determine whether adding LET to primary ACLR improves various outcomes, there is strong evidence that LET effectively reduces laxity in the lateral compartment, as demonstrated by stress radiography. 28 29

In the biomechanics study, there is still a controversy regarding ACLR + LET with the modified Lemaire technique. A laboratory study 10 with a fresh frozen cadaver found that this technique might have overconstrained knee kinematics. However, a pilot study by Di Benedetto et al. 30 on 16 patients aged 21 to 37 years who underwent ACLR + LET revealed reacquisition of sagittal knee stability and gait dynamics to the preoperative level. These findings are also supported by a meta-analysis by Feng et al., 31 who reported that, in 1,745 patients, ACLR + LET provided reduced pivot-shif,t with an odds ratio of 0.48 (95% CI: 0.31 to 0.74), and better graft failure rate, with an odds ratio of 0.34 (95%CI: 0.20 to 0.55).

As a limitation of the present study, there is still a lack of raw data to make a more comprehensive functional outcome analysis. Therefore, future studies with large samples might be needed to find better evidence regarding the effectiveness of ACLR combined with LET through the modified Lemaire technique.

## Conclusion

The combination of LET through the modified Lemaire technique and ACLR showed a reliable result to minimize the rate of graft rerupture, as well as superiority in terms of clinical outcomes compared with isolated ACLR due to its role in improving knee stability.
