# Supplementary material for: Combined Anterior Cruciate Ligament Reconstruction (ACLR) and Lateral Extra-articular Tenodesis through the Modified Lemaire Technique versus Isolated ACLR: A Meta-analysis of Clinical Outcomes
Source: Rev Bras Ortop (Sao Paulo). 2024 Apr 10;59(2):e180–8. doi: 10.1055/s-0044-1785492 (PMC11006520; doi:10.1055/s-0044-1785492)
Supplement: Related texts

# Research article: Reconstrução combinada do ligamento cruzado anterior (RLCA) e tenodese extra-articular lateral pela técnica de Lemaire modificada *versus* RLCA isolada: Uma metanálise dos resultados clínicos

**Keywords:** reconstrução do ligamento cruzado anterior, instabilidade articular, articulação do joelho, tenodese, resultado do tratamento

## Abstract

**Objetivo** A tenodese extra-articular lateral (TEL) foi proposta para resolver a instabilidade rotatória após a reconstrução do ligamento cruzado anterior (RLCA). Esta metanálise teve como objetivo comparar os resultados clínicos da RLCA e da RLCA com TEL por meio da técnica de Lemaire modificada.

**Materiais e Métodos** Esta metanálise foi feita de acordo com a declaração dos Itens Principais para Relatar Revisões Sistemáticas e Metanálises (Preferred Reporting Items for Systematic Reviews and Meta-Analysis, PRISMA, em inglês). A pesquisa bibliográfica foi realizada nos bancos de dados PubMed, EBSCOHost, Scopus, ScienceDirect e WileyOnline. Dos estudos incluídos foram extraídas informações sobre as características do estudo, a taxa de falha (falha clínica ou do enxerto) como resultado primário, e o escore funcional como resultado secundário. Foram feitas comparações entre os pacientes submetidos apenas à RLCA (grupo RLCA) e à RLCA e TEL pela técnica de Lemaire modificada (grupo RLCA + TEL).

**Resultados** Foram avaliados 5 estudos que incluíam 797 pacientes. O grupo RLCA + TEL apresentou um risco menor de falha e menor taxa de rerruptura do que o grupo RLCA (razão de risco [RR] = 0,44; intervalo de confiança de 95% [IC95%] 0,26 a 0,75; I ^2^ = 9%; *p* = 0,003). O grupo RLCA + TEL obteve pontuações maiores no Escore de Desfechos de Osteoartrite e Lesão no Joelho (Knee Injury and Osteoarthritis Outcome Score, KOOS, em inglês) com relação aos seguintes desfechos: dor, atividades cotidianas (AC), esportes, e qualidade de vida (QV), com diferenças médias de 0,20 (IC95%: 0,10 a 0,30; I ^2^ = 0%; *p* < 0,0001), -0.20 (IC95%: -0,26 a-0,13; I ^2^ *=* 0%; *p* < 0,00001), 0,20 (IC95%: 0,02 a 0,38; I ^2^ = 0%; *p* = 0,03) e 0,50 (IC95%: 0,29 a 0,71; I ^2^ = 0%; *p* < 0,00001), respectivamente, quando comparado com o grupo RLCA.

**Conclusão** O acréscimo de TEL pela técnica de Lemaire modificada à RLCA pode melhorar a estabilidade do joelho devido à menor taxa de rerruptura do enxerto e à superioridade dos resultados clínicos.

**Nível de evidência** I.

## Introdução

As rupturas do ligamento cruzado anterior (LCA) estão entre as lesões mais comumente estudadas em pesquisas ortopédicas,e estima-se que a sua incidência varia de 30 a 78 casos por 100 mil pessoas por ano. 1 Após a reconstrução do LCA (RLCA), 61% a 89% dos atletas retornam com sucesso aos esportes, geralmente de 8 a 18 meses após a reconstrução, dependendo do nível de jogo. 1 Em algumas condições, há a possibilidade de ocorrer uma nova ruptura, o que pode ser devastador. A taxa relatada de rerruptura do LCA varia de 1% a 11%. As rerrupturas podem ser causadas por novas lesões traumáticas, falha biológica do enxerto ou erros técnicos cirúrgicos. 1 2

O tratamento da lesão do LCA em pacientes com maior risco de rerruptura permanece controverso. Foi demonstrado que os fatores de risco para a ruptura do enxerto incluem pacientes mais jovens (< 20 anos), aqueles com hipermobilidade generalizada e hiperextensão fisiológica do joelho e aqueles que retornam a esportes de alto risco (pivotantes). 3 Além disso, Saita et al. 4 demonstraram que a hiperextensão do joelho e um côndilo lateral pequeno estão associados a uma maior instabilidade rotatória anterolateral. É difícil manejar a instabilidade rotatória em pacientes que continuam a apresentar um deslocamento de pivô positivo após a RLCA isolada. A literatura mostra 3 5 6 7 que as técnicas de reconstrução de MacIntosh, Lemaire e do ligamento anterolateral (LAL) resolvem a instabilidade rotatória anterolateral. Descobriu-se que a reconstrução do LAL reduziu a taxa de falha do enxerto em uma grande série de pacientes com dois anos de acompanhamento. 8 A técnica de Lemaire modificada demonstrou ter uma baixa taxa de complicações e provocar redução da instabilidade de deslocamento do pivô. 6

Um dos motivos que favorecem a tenodese extra-articular lateral(TEL) em vez da reconstrução do LAL é a evidência que indica que a reconstrução do LAL pode sobrecarregar a articulação lateral e não ser tão vantajosa mecanicamente na resistência à rotação. 9 10 O objetivo da realização da TEL é diminuir a taxa de rerruptura, ao proporcionar mais estabilidade à articulação do joelho. Um estudo de coorte realizado por Cavaignac et al. 11 mostrou que a RLCA com TEL apresentou melhor maturidade do enxerto em exames de ressonância magnética (RM) após um ano do procedimento. Mayr et al. 12 concentraram-se na técnica de Lemaire modificada, que foi recentemente realizada na TEL, e mostraram que ela pode diminuir a tensão no enxerto e a frouxidão rotacional residual, melhorando assim os resultados clínicos. Portanto, realizamos uma metanálise para determinar o impacto da RLCA e da TEL com a técnica de Lemaire modificada em comparação com a RLCA em pacientes com ruptura do LCA em termos de taxa de rerruptura e do resultado clínico. O objetivo deste estudo foi determinar o resultado cirúrgico da RLCA com TEL pela técnica modificada de Lemaire para ruptura do LCA, que é representado pela taxa de rerruptura e pelo resultado clínico.

## Materiais e Métodos

## Estratégia de pesquisa

Realizamos uma revisão sistemática e uma metanálise com base na declaração dos Itens Principais para Relatar Revisões Sistemáticas e Metanálises (Preferred Reporting Items for Systematic Reviews and Meta-Analysis, PRISMA, em inglês). 13 O protocolo do estudo foi registrado no Open Science Framework. A pesquisa bibliográfica foi realizada em junho de 2022 em vários bancos de dados, incluindo PubMed, EBSCOHost, Scopus, ScienceDirect e WileyOnline, e centrada na estratégia de População, Intervenção, Controle e Resultado (Population, Intervention, Control, and Outcome, PICO, em inglês). A população consistiu em pacientes com ruptura do LCA. A intervenção foi a RLCA e a TEL pela técnica de Lemaire modificada. A RLCA isolada foi o parâmetro de comparação. Os resultados avaliados foram a taxa de rerruptura como resultado primário e as medidas de resultado relatadas pelo paciente ( *patient-reported outcome measures* , PROMs, em inglês]) e o escore funcional como resultados secundários.

## Seleção do estudo

Os critérios de exclusão foram estudos em animais, casos de revisão da RLCA, reconstrução concomitante do ligamento cruzado posterior (LCP) ou do menisco, condição congênita ou neoplasia subjacente, RLCA com reconstrução do LAL, pacientes submetidos a tratamento farmacológico, tratamento nutricional, fisioterapia ou reabilitação isolada, e RLCA com TEL que não utilizou a técnica de Lemaire modificada. Somente foram incluídos estudos publicados em inglês nos últimos vinte anos.

## Avaliação da qualidade e avaliação do risco de viés

Dois autores (ED e LC) realizaram a identificação, seleção e extração de dados. A avaliação da qualidade foi realizada por outros dois autores (MS, IJA). Diferenças de opinião entre os dois revisores foram resolvidas por meio de reavaliação e discussão com outro autor (EK). Os estudos selecionados fora, avaliados por meio das ferramentas de avaliação crítica do Joanna Briggs Institute. 14

## Extração e análise de dados

Os dados extraídos dos estudos incluídos foram características como autor e ano de publicação, local, desenho, características da amostra (idade, gênero, tipo de lesão), falha (falha clínica ou do enxerto) e resultado (pontuação no Escore de Desfechos de Osteoartrite e Lesão no Joelho [Knee Injury and Osteoarthritis Outcome Score, KOOS, em inglês]), resultado funcional, resultado clínico). Os estudos foram avaliados qualitativa e quantitativamente por meio do programa Review Manager (RevMan, The Cochrane Collaboration, Londres, Reino Unido), versão 5.4. Um modelo de efeito aleatório foi usado para calcular a proporção combinada de cada estudo com base na heterogeneidade. O teste do I ao quadrado (I ^2^ ) da Cochrane foi realizado para determinar a heterogeneidade. Os resultados dos estudos foram apresentados em um gráfico de floresta com razão de risco (RR) agrupada.

## Resultados

Na triagem inicial, foram encontrados163 estudos ( Fig. 1 ). Entre os dez estudos restantes, dois não tinham um resultado primário (taxa de sucesso), 12 15 um estudo 16 incluiu pacientes esqueleticamente imaturos e outro 17 não tinha controle adequado. Por fim, encontramos cinco estudos 18 19 20 21 22 elegíveis para as análises qualitativa e quantitativa após a aplicação das estratégias de busca. Dois estudos eram ensaios clínicos randomizados (RCTs) 18 19 e três eram estudos de coorte. 20 21 22

**Fig. 1:** Fluxograma PRISMA.

A avaliação dos estudos usando as ferramentas de avaliação crítica do Joanna Briggs Institute mostrou que todos foram considerados bons em termos de qualidade metodológica e ausência de possibilidade de viés em seu projeto, condução e análise ( Tabela 1 ). A Tabela 2 mostra as características do estudo, inclusive os detalhes intraoperatórios. A Tabela 3 mostra os parâmetros de resultados medidos para cada estudo.

Neste estudo, constatou-se que a taxa de risco de falha foi menor no grupo RLCA + TEL pela técnica de Lemaire modificada do que no grupo RLCA, com baixa heterogeneidade entre os estudos (RR = 0,44; intervalo de confiança de 95% [IC95%]: 0,26 a 0,75; I ^2^ = 9%; *p* = 0,003) ( fig. 2 ).

**Tabela 1:** Resultados da avaliação crítica dos estudos selecionados

Estudo	Itens relacionados às ferramentas de avaliação crítica do Joanna Briggs Institute												
	1	2	3	4	5	6	7	8	9	10	11	12	13
Castoldi et al. 18 (2020)	S	S	S	ND	ND	S	S	S	S	S	S	S	S
Getgood et al. 19 (2020)	S	S	S	ND	ND	S	S	S	S	S	S	S	S
Eggeling et al. 21 (2022)	S	S	S	ND	ND	S	S	S	S	S	S	S	S
Rowan et al. 22 (2019)	S	S	S	S	S	S	S	S	S	S	S		
Dejour et al. 20 (2013)	S	S	S	ND	ND	S	S	S	S	S	S		

Abreviaturas: ND, não available; S, sim.

**Tabela 2:** Características dos estudos incluídos

Estudo	Tipo	Nível de evidência	Número de pacientes						Idade média (anos)		Média/valor mínimo do acompanhamento (anos)	Tempo médio da lesão à cirurgia (meses)		Enxerto		Método de tensionamento	Fixação da TEL	Fixação do LCA
			TEL			Não TEL			TEL	Não TEL		TEL	Não TEL	LCA	TEL			
			M	F	T	M	F	T										
Castoldi et al. 18 (2020)	ECCR	2	47	13	60	43	18	61	26.2	25.9	19.4	NR	NR	OPTO	G	30 graus de flexão do joelho	Fixação por sutura	Lados femoral/tibial: parafuso de interferência absorvível
Getgood et al. 19 (2020)	ECCR	2	151	155	306	151	161	312	19.1	18.8	2	9.3	8.1	I	BIT	60-70 graus de flexão do joelho	Grampo de fixação farpado de Richards	Lado femoral: fixação suspensória cortical femoral; lado tibial: parafuso de interferência tibial
Eggeling et al. 21 (2022)	C	3	13	10	23	35	20	55	33.3	31.9	2.4	NR	NR	OPTO, I, Q	BIT	45 graus de flexão do joelho	Fixação por sutura	Ladod femoral/tibial: parafuso de interferência
Rowan et al. 22 (2019)	C	3	34	21	55	120	98	218	26	33	2	NR	NR	ST + G	BIT	30 graus de flexão do joelho	Grampo de fixação com espiga	Lados femoral/tibial: parafuso de interferência absorvível
Dejour et al. 20 (2012)	C	3	20	5	25	17	8	25	21.4	27.5	2	10.78	12.96	OPTO	G	Extensão total	Fixação por sutura	Lados femoral/tibial:parafuso de interferência

Abreviaturas: BIT, banda iliotibial; C, coorte; ECCR, ensaio clínico randomizado e controlado; F, feminino; G, grácil; I, isquiotibiais; LCA, ligamento cruzado anterior; M, masculino; NR, não relatado; OPTO, osso-patela tendão-osso; Q, quadríceps; ST, semitendinoso; T, total; TEL, tenodese extra-articular lateral.

**Tabela 3:** Resultados dos estudos incluídos

Estudo	Falha (%TEL vs. %controle)	Resultado								
		Pontuação de dor do KOOS	Pontuação de sintomas do KOOS	Pontuação de AC do KOOS	Pontuação de esportes do KOOS	Pontuação de QV do KOOS	Pontuação de Tegner	Pontuação do Comitê Internacional de Documentação do Joelho	Pontuação de Lysholm	Pontuação de Marx
Castoldi et al. 18 (2020)	Falha do enxerto: 5/38 (13%) vs. 12/42 (29%)	−	−	−	−	−	−	82,4 ± 11,2 vs. 81,1 ± 14,38	−	−
Getgood et al. 19 (2020)	Falha clínica: 72/291 (25%) vs. 120/298 (40%) (RR: 0,38; IC95%: 0,21–0,52; *p* = 0,0001).	92,1 ± 0,6 vs. 91,9 ± 0,6	84,7 ± 0,8 vs. 84,6 ± 0,8	97 ± 0,4 vs. 97,2 ± 0,4	85,3 ± 1,1 vs. 85,1 ± 1,1	63,8 ± 18,9 vs. 58,4 ± 19,7	−	87,3 ± 0,8 vs. 86,8 ± 0,8	−	12,1 ± 5,5 vs. 12,7 ± 4,7
	Falha do enxerto: 11/291 (4%) vs. 34/298 (11%) (RR: 0,67; IC95%: 0,36–0,83; *p* = 0,001)						−	−	−	−
Eggeling et al. 21 (2022)	Falha clínica: 3/23 (13%) vs. 6/55 (10,9%)	87,9 ± 14,6 vs. 87,9 ± 14,1	87,6 ± 15,4 vs. 87,3 ± 14,8	95,2 ± 8,2 vs. 93 ± 10	72,6 ± 25,9 vs. 76 ± 22,7	75,4 ± 1,3 vs. 74,9 ± 1,3	5,7 ± 1,3 vs. 5,9 ± 1,5	80,1 ± 14,9 vs. 77,5 ± 16,2	81,9 ± 14,2 vs. 83,8 ± 14,5	−
Rowan et al. 22 (2019)	Falha clínica: 0/46 (0%) vs. 13/125 (10,4%)	−	−	−	−	−	8,04 ± 1,35 vs. 75,4 ± 1,35	−	98 ± 11,2 vs. 90 ± 16,2	−
Dejour et al. 20 (2012)	Falha clínica: 0/25 (0%) vs. 2/25 (8%)	−	−	−	−	−	−	−	−	−

**Abreviaturas:** AC, atividades cotidianas; IC95%, interval de confiança de 95%; KOOS, Escore de Desfechos de Osteoartrite e Lesão no Joelho (Knee Injury and Osteoarthritis Outcome Score, em inglês); QV, qualidade de vida; RR, razão de risco; TEL, tenodese extra-articular lateral; vs., versus.

**Fig. 2:** Razão de risco de falha do grupo RLCA + TEL pela técnica de Lemaire modificada e do grupo RLCA.

A metanálise mostrou uma superioridade do grupo RLCA + TEL pela técnica de Lemaire modificada com relação aos seguintes desfechos do KOOS: dor, atividades cotidianas (AC), esportes e qualidade de vida (QV), com diferenças médias de 0.20 (IC95%: 0,10 a 0,30; *p* < 0,0001), -0,20 (IC95%: -0,26 a -0,13; *p* < 0,00001), 0,20 (IC95%: 0,02 a 0,38; *p* = 0,03) e 0,50 (IC95%: 0,29 a 0,71; *p* < 0,00001), respectivamente. Entretanto, não houve diferença significativa nas pontuações de sintomas do KOOS entre os grupos, com uma diferença média de 0,10 (IC95%: -0,03 a 0,2; *p* = 0,13). Tampouco houve diferenças entre os grupos com relação às pontuações na Escala de Atividade de Tegner (Tegner Activity Scale, TAS em inglês) e na Escala de Pontuação do Joelho de (Lysholm Knee Scoring Scale, LKSS, em inglês), com diferenças médias de 0,19 (IC95%: -0,49 a 0,87; *p* = 0,58) e 3,45 (IC95%: -6,22 a 13,22; *p* = 0,48), respectivamente. Houve diferença significativa na pontuação no Formulário Subjetivo de Joelho do International Knee Documentation Committee (IKDC), com diferença média de 0,70 (IC95%: 0,57 a 0,83; *p* < 0,00001). Foi encontrada baixa heterogeneidade nas pontuações no KOOS e no IKDC, mas foi encontrada alta heterogeneidade nas pontuações de Tegner e Lysholm ( Fig. 3 ).

**Fig. 3:** Gráfico de metanálise do resultado secundário dos estudos incluídos.

## Discussão

Os achados mais importantes desta pesquisa foram que, em comparação com o grupo RLCA, o RLCA + TEL pela Lemaire modificada teve uma taxa de falha menor e superioridade significativa em relação ao resultado funcional com base nas diferenças médias nos domínios de dor, AC, esportes e QV.

Quando comparado com o grupo RLCA, o grupo RLCA + TEL pela técnica de Lemaire modificada apresentou uma taxa de falha menor (RR = 0,44; I ^2^ = 9%; *p* = 0,003). A RLCA + TEL pela técnica de Lemaire modificada mostrou superioridade significativa em relação ao resultado funcional com base nas diferenças médias nos domínios de dor, AC, esportes e QV medidos pelas pontuações no KOOS ( *p* < 0,00001; *p* < 0,03; *p* < 0,00001; e *p* < 0,00001, respectivamente) e no IKDC ( *p* < 00001).

A estabilidade rotacional não foi recuperada com a RLCA isolada em uma determinada população. 23 Portanto, foram necessários procedimentos intra e extra-articulares para melhorar a estabilidade do LCA, melhorando assim a capacidade de praticar esportes nessa população. Sabe-se que a TEL era um dos procedimentos extra-articulares para preservar a estabilidade do joelho. Na et al. 23 compararam a RLCA isolada à RLCA combinada com procedimentos extra-articulares anterolaterais, e observaram que ambas as técnicas melhoraram os graus de mudança do pivô e as taxas de falha do enxerto. No entanto, no grupo RLCA + TEL, houve um risco maior de rigidez do joelho e de eventos adversos. 23 Esses achados explicam as pontuações significativamente melhores no KOOS e no IKDC no grupo RLCA + TEL pela técnica de Lemaire modificada.

Vários procedimentos de TEL, a saber, Lemaire, MacIntosh e reconstrução do LAL, são as opções para manejar a instabilidade rotatória. No entanto, em um estudo cinemático publicado por Inderhaug et al. 10 em 2017, constatou-se que a reconstrução do LAL é um procedimento de restrição insuficiente. Em comparação com a reconstrução do LAL, a técnica Lemaire modificada demonstrou resultar em uma baixa taxa de complicações e provocar redução da instabilidade de deslocamento do pivô. A técnica de Lemaire modificada também resultou em boa sobrevida do enxerto e boas PROMs em uma população de alto risco, 1 o que pode sugerir que a TEL é uma técnica eficaz para restaurar a estabilidade da articulação em um joelho com características adicionais de frouxidão. 2 10

Uma metanálise, Onggo et al. 24 compararam RLCA e RLCA + TEL por qualquer método, com a inclusão de estudos com um mínimo de dois anos de acompanhamento. Eles descobriram que houve maior estabilidade (RR = 0,59; IC95%: 0,39 a 0,88) e melhores resultados clínicos no grupo RLCA + TEL, demonstrados pelas diferenças médias nas pontuações no IKDC e na escala de Lysholm, com diferenças médias de 2,31 (IC95%: 0,54 a 4,09) e 2,71 (IC95%: 0,68 a 4,75), respectivamente. Além disso, houve menor probabilidade de rerruptura do enxerto no grupo RLCA + TEL, com uma RR de 0,31 (IC95%: 0,17 a 0,58). 24 Uma revisão sistemática de braço único envolvendo 851 pacientes submetidos a RLCA + TEL, feita por Grassi et al., 25 mostrou resultados favoráveis nas pontuações no KOOS, com 74% dos pacientes voltando a realizar suas atividades esportivas anteriores, juntamente com taxas de complicação e falha de 8,0% e 3,6%, respectivamente.

O RLCA + TEL também foi considerado seguro para os pacientes. Um estudo de Feller et al. 26 descreveu que, no acompanhamento de 12 meses, somente ocorreu ruptura do enxerto relacionada ao contato em um paciente, representando 4% do total. Foram observadas 2 lesões adicionais do LCA no joelho oposto, representando 9% dos casos, sendo uma delas uma ruptura do enxerto do LCA 11 meses após a cirurgia e outra, aos 22 meses. Além disso, um incidente separado de ruptura do enxerto de LCA contralateral ocorreu no acompanhamento de 26 meses. 26 Foram levantadas preocupações sobre o potencial de restrição excessiva do compartimento lateral do joelho e o desenvolvimento subsequente de osteoartrite do compartimento lateral em relação ao TEL. No entanto, uma metanálise realizada por Devitt et al. 27 fornece evidências sólidas de que o acréscimo da TEL reduz o movimento do compartimento lateral. Estudos biomecânicos apoiam esses achados clínicos, e mostram que tanto a reconstrução anatômica do LAL quanto os procedimentos de TEL podem restringir excessivamente o compartimento lateral. Por outro lado, uma revisão sistemática recente indicou que o acréscimo da TEL à RLCA não aumenta as taxas de osteoartrite em longo prazo. Embora não haja evidências suficientes para determinar se o acréscimo da TEL à RLCA primária melhora vários resultados, há evidências sólidas de que a TEL é eficaz na redução da frouxidão no compartimento lateral, conforme demonstrado pela radiografia de estresse. 28 29

No estudo biomecânico, ainda há uma controvérsia em relação à RLCA + TEL pela técnica Lemaire modificada. Um estudo de laboratório 10 com um cadáver congelado ainda fresco constatou que essa técnica pode ter restringido excessivamente a cinemática do joelho. No entanto, um estudo piloto realizado por Di Benedetto et al. 30 em 16 pacientes com idades entre 21 e 37 anos submetidos à RLCA + TEL revelou a reaquisição da estabilidade sagital do joelho e da dinâmica da marcha no nível anterior ao da cirurgia. Esses achados também são apoiados por uma metanálise realizada por Feng et al., 31 que relataram que, em 1.745 pacientes, a RLCA + TEL proporcionou redução do mudança do pivô, com uma razão de chances de 0,48 (IC95%: 0,31 a 0,74) e melhor taxa de falha do enxerto, com uma razão de chances de 0,34 (IC95%: 0,20 a 0,55).

Uma limitação deste estudo é o fato de que ainda há escassez de dados brutos para se fazer uma análise mais abrangente dos resultados funcionais. Portanto, talvez sejam necessários estudos futuros com amostras grandes para que se encontrem melhores evidências sobre a eficácia da RLCA e da TEL pela técnica de Lemaire modificada.

## Conclusão

A combinação da TEL pela técnica de Lemaire modificada com a RLCA mostrou um resultado confiável em termos da redução da taxa de rerruptura do enxerto, além de superioridade nos resultados clínicos em comparação com a RLCA isolada, devido ao seu papel na melhora da estabilidade do joelho.
